# Genomic and functional characterization of the *cinnamic acid 4-hydroxylase* gene family reveals candidate genes involved in rind texture modulation in watermelon

**DOI:** 10.3389/fpls.2026.1887090

**Published:** 2026-06-30

**Authors:** Mingsen Cui, Xiya Sun, Liyuan Yang, Sikandar Amanullah, Chaonan Wang, Xuezheng Wang, Zuyun Dai, Zhongzhou Yang, Tiantian Yang, Huilin Wang

**Affiliations:** 1 College of Horticulture, Xinjiang Agricultural University, Urumqi, China; 2 Xinjiang Special Melon and Fruit Variety Improvement and Logistics Transportation Joint Research Center, Xinjiang Agricultural University, Urumqi, China; 3 Department of Horticultural Science, North Carolina State University, Mountain Horticultural Crops Research and Extension Center, Mills River, NC, United States; 4 College of Horticulture, Northeast Agricultural University, Harbin, China; 5 China-Costa Rica Belt and Road Joint Laboratory on Fruit and Vegetable Bio-Breeding and Intelligent Technology, Anhui Jianghuai Horticulture Technology Co., Ltd., Hefei, China; 6 Key Laboratory of Fruit and Vegetable Germplasm Innovation and Utilization in Jianghuai Region, Ministry of Agriculture and Rural Affairs, Hefei, China

**Keywords:** *C4H* gene family, gene expression, lignin biosynthesis, rind hardness, watermelon

## Abstract

Rind hardness is a significant horticultural trait that directly determines sensory quality and postharvest shelf life. Lignin deposition in the stone cell layer is the primary biochemical determinant of fruit rind, yet the genetic regulatory basis of lignin biosynthesis in watermelon has been studied less. Cinnamic acid 4-hydroxylase (C4H) is a rate-limiting enzyme in the phenylpropanoid metabolic cycle, essential for lignin biosynthesis in plants and the formation of robust fruit rinds. However, the in-depth genetic regulatory basis of rind hardness in many fruits has not been investigated. Herein, we conducted the first genome-wide bioinformatics analysis to identify the characterization of the *C4H* gene family and to explore its potential functions through physiological and molecular experiments in watermelon. The retrieved results exposed a total of 4 *ClC4H* members, and phylogenetic analysis classified them into two closely associated branches: Class I and Class II. The gene promoter analysis revealed that the upstream region of the *ClC4H* gene not only contains a large number of essential core elements for basic transcription but also is densely enriched with cis-acting elements that respond to light, various hormones (such as ABA, auxin, etc.), and non-biological stresses, forming a complex transcriptional regulatory network. Transcriptomic and physiological analysis showed that candidate ClC4H1 and ClC4H2 genes exhibited elevated expression during early fruit rind development, which tended to coincide with higher endogenous lignin content. The predicted protein-protein interaction and molecular docking suggested that ClC4H1 might form an interaction network with ClPAL and ClCCR2-like to participate in lignin synthesis. The RT-qPCR-based relative expressions of the candidate genes also validated the differentiated lignin biosynthesis at different developmental stages of rind texture in contrasted watermelon materials; however, subcellular localization confirmed that ClC4H1 is located in the endoplasmic reticulum. These findings provide important candidate genes and a theoretical basis for using molecular breeding techniques to improve the texture of watermelon fruit.

## Introduction

The phenylpropanoid metabolic pathway is an important branch of plant secondary metabolism, providing a large amount of lignin, flavonoids, anthocyanins, and various phenolic substances for plant life activities (Vogt, 2010; Fraser and Chapple, 2011). These substances not only affect the lignification process of cell walls during plant growth and development but also have multiple functions, such as responding to biological and abiotic stresses and cell signal transduction. For example, lignin is formed by the cross-linking of high-molecular-weight polymers through covalent bonds such as *β*-O-4, *β*-*β*, *β*-5, and 5–5 and connects with cellulose fibrils to strengthen plant tissues. It can not only ensure the long-distance transportation of water and minerals but also form a physical barrier to resist biological and abiotic stresses (Dixon and Paiva, 1995; Cheynier et al., 2013). Therefore, using bioinformatics methods to systematically analyze the functional characteristics of key enzyme gene families in this metabolic pathway has important theoretical significance for elucidating the formation mechanism of plant complex traits and improving the adaptability of crops to stress.

Cinnamate-4-hydroxylase (C4H), a rate-limiting and hub enzyme in the early stage of the phenylpropanoid pathway, plays a crucial role in determining whether the carbon flow fixed by photosynthesis is more allocated to the lignin secondary metabolites that build the cell wall or to the active substances, such as flavonoids, that perform physiological regulatory functions (Winkel-Shirley, 2001; Ehlting et al., 1999). The bioinformatic studies have found that C4H belongs to the cytochrome P450 superfamily (CYP73A subfamily), and its protein is anchored to the endoplasmic reticulum membrane through the hydrophobic structure at the N-terminus. It often forms a dynamic multi-enzyme complex with the upstream phenylalanine deaminase (PAL) and the downstream 4-caffeoyl-CoA ligase (4CL) on the surface of the endoplasmic reticulum membrane. This “metabolic channel” effect helps to efficiently guide the directed transformation of substrates, but the precise assembly, regulatory mechanism, and conservation of this complex in different species still need to be further elucidated (Achnine et al., 2004; Chen et al., 2011).

The systematic bioinformatic analysis of the *C4H* gene family has been performed in multiple plant species due to the effective development of genome sequencing projects. E.g., two *C4H* genes were dissected in the *Vanilla planifolia* genome, and their internal promoter regions showed abundant cis-regulatory elements related to light and stress-related response. The tissue expression profile analysis indicated that they were mainly expressed in flowers, depicting a relatively lower expression in other tissues (Kaur et al., 2025). A total of four *C4H* members were identified in soybean plants that were mainly divided into two major evolutionary branches. Yeast heterologous expression confirmed that three of them have enzymatic activity and can hydroxylate trans-cinnamic acid to varying degrees [10]. A latest study on rice (*Oryza sativa* L.) indicated that although *C4H* plays an important role in the core lignin synthesis, its functional loss can be significantly compensated by a unique “PTAL” pathway specific to grass plants, starting from tyrosine, to maintain normal lignin synthesis and cell wall integrity. This discovery provided a new perspective for understanding the differences in lignin synthesis regulation between monocotyledonous and dicotyledonous plants (Supatmi et al., 2025).

Furthermore, numerous crop studies [e.g., tea trees (Zhang et al., 2024) and honeysuckle (Zeng et al., 2025)] have likewise indicated that the expression of *C4H* genes is considerably connected with the biosynthesis of chlorogenic acid and catechins, emphasizing its potential application. These studies collectively demonstrated that the *C4H* gene family has conserved phylogenetic and functional diversity in plants. Its members not only play key roles in the core pathway of phenylpropanoid metabolism, especially lignin synthesis, but also undergo functional differentiation in different species and tissues (Hamberger et al., 2011; Xu et al., 2024).

A genome-wide analysis method for evolutionary biology evaluation is called “gene family analysis” and has been widely applied to characterize the groups of homologous genes that share a common ancestral gene, which are typically conserved between species or within a genome (Demuth and Hahn, 2009; Lespinet et al., 2002). It is a fundamental technique in the modern genomics research trends and proposes an organized framework for connecting gene evolution, structure, and major function across biological systems (Fitch, 2000; Koonin, 2005). Over the last decade, genome-wide bioinformatics analysis of gene families has become a quick and proficient method for pinpointing potential functional genes governing a variety of crop-specific traits, such as development, stress tolerance, and yield-related traits (Cannon et al., 2004; Xu et al., 2012).

Watermelon is an important economic fruit crop worldwide. The texture of the fruit rind directly affects the commodity value and commercial industry efficiency. Lignin, as the key determinant of the lignification of the stone cell layer in the fruit rind, directly influences the firmness quality, storage life of the fruit, and transportation tolerance (Zhang et al., 2022; Zheng et al., 2026). Recently, the *C4H* gene has been proven to be one of the key genes regulating the lignification of fruit stone cells in pears (Li et al., 2020) and peaches (Hu et al., 2011), and its expression level is significantly positively correlated with the accumulation of lignin. Gene defects can lead to significant changes in the composition and content of lignin. However, the *C4H* gene family in watermelon has not yet been systematically identified at the whole genome level. The number of its members, molecular characteristics, evolutionary relationships, and expression patterns in different developmental periods and tissues of the fruit, as well as the response mechanisms to adverse stress, remain unknown. This knowledge gap greatly limits our ability to improve the fruit quality and plant resistance through molecular means.

Hence, our study is the first systematic analysis of the *C4H* gene family, aiming to screen and predict potential key *C4H* genes that regulate lignin biosynthesis in watermelon. This analysis is based on integrated genome-wide bioinformatics and gene expression profiling across different tissues and key developmental periods of germplasm that show significant differences in lignin accumulation in the fruit rind. We believe that our study provides preliminary genomic clarification about the biological characteristics of the *C4H* gene family, lays the foundation for in-depth analysis of the complex molecular regulatory network of lignin synthesis, and provides potential genetic resources and a theoretical basis for molecular breeding in watermelon for better fruit quality.

## Materials and methods

### Research materials

Two contrasted experimental materials of watermelon (WRH, watermelon fruit with a hard fruit rind, gene bank accession number ZXG00430, and WRS, watermelon fruit with a soft fruit rind, gene bank accession number ZXG00812) were utilized in this study. These materials are self-fertilized, high-generation varieties of watermelons. The seeds were provided by the West Melon and Watermelon Research Group of the Horticulture College of Northeast Agricultural University, Harbin, and planted at the Sanping Teaching and Practice Base of Xinjiang Agricultural University (87.35° E, 43.94° N). The crop plants were cultivated in an open field with a planting pattern of 150 cm of row spacing and 30 cm of plant spacing; however, field management practices were uniformly carried out using the double-vine pruning method to leave a single fruit per plant.

### Genomic sequencing

The young fresh leaves were sampled from the contrasted watermelon materials (WRH and WRS), placed in 2 mL centrifuge tubes, quickly cryo-frozen with liquid nitrogen, and then crushed into fine powder just before the experiment. The high-quality purified DNA was extracted using the cetyltrimethylammonium bromide (CTAB) extraction method (Allen et al., 2006), and the quality and concentration of purified DNA were quantified using spectrophotometry and agarose gel electrophoresis. The high-quality genomic DNA was utilized for whole-genome resequencing at high sequencing depth (>25x) using the Illumina HiSeq 2500 system by China Shenzhen BGI Technology Co., Ltd. The uploaded genome resequencing data can be downloaded from the National Center for Biotechnology Information (NCBI) with the accession number (PRJNA915415; https://www.ncbi.nlm.nih.gov/bioproject/?term=PRJNA915415) (Yang et al., 2023).

### Transcriptomic sequencing

For the transcriptomic sequencing analysis, three fruits with similar-sized growth were harvested on different days after pollination (1, 14, and 28 DAP). A total of 1 gram of fruit rind tissue was cut and weighed from three parts of the fruits of both watermelon materials (WRH and WRS) following three replications, then placed in 25 mL centrifuge tubes, and marked with their respective times. The actual experimental replications were labeled for both watermelon experimental materials as follows: WRH-1d (1, 2, 3), WRH-14d (1, 2, 3), WRH-28d (1, 2, 3), WRS-1d (1, 2, 3), WRS-14d (1, 2, 3), and WRS-28d (1, 2, 3).

The high-quality and purified total RNA was isolated from replicated fruit rind tissues by the QIAGEN RNeasy Mini Kit or Plus Kit (QIAGEN, Hilden, Germany) using silica-column technology for consistent results; the concentration and purity of isolated RNA were measured through a spectrophotometer, and integrity was assessed by agarose gel electrophoresis. RNA sequencing was completed at Wuhan Maiwei Metabolic Biotechnology Co., Ltd. All the uploaded raw sequencing data can be downloaded from the Online Sequence Read Archive (SRA) database of the National Center for Biotechnology Information (NCBI) with the access number (PRJNA1096440; https://www.ncbi.nlm.nih.gov/bioproject/PRJNA1096440/). Herein, we identified the differential expression genes (DEGs) using DESeq2 (v1.34.0) based on a negative binomial model; the false discovery rate (FDR) was controlled via the Benjamini–Hochberg procedure; genes with an absolute |log_2_ fold change| > 1 and an FDR-adjusted p-value < 0.05 were considered significantly differentially expressed.

### Identification of *ClC4H* gene family members and protein characteristics

The complete genomic sequence, CDS sequence, protein sequence, and annotation file of the watermelon genome (97103, v2) were downloaded (accessed on January 5, 2026) from the CuGenDB database (Zheng et al., 2019). The candidate gene family members were identified by searching for the keyword “Cytochrome P450 family cinnamate 4-hydroxylase” and the homologous alignment sequence of the published protein sequence [AtC4H (AT2G30490.1)] was searched through the BLAST function. The Hidden Markov model (HMM) of the cytochrome P450 protein domain (PF00067) from the Pfam database was downloaded (Paysan-Lafosse et al., 2025).

The HMM-search function of HMMER 3.0 software (Eddy, 2011) was used to search the protein sequence of watermelon for the PF00067 HMM, with the threshold set at E < 1×10^-5^. All the obtained candidate genes were taken as the union set, and their presence of the Cytochrome P450 protein domain was queried using the databases of NCBI-CDD (Yang et al., 2020) and SMART, a simple modular architecture research tool (Schultz et al., 1998). Genes without the Cytochrome P450 protein domain and repetitive genes were deleted to determine the final family members of the watermelon *ClC4H* gene.

The protein molecular mass, isoelectric point, and amino acid length of the watermelon *ClC4H* gene family members were predicted using the ProtParam tool in the ExPASY platform (Wilkins et al., 1999). The protein secondary structure prediction function in Network Protein Sequence Analysis was used, and the SOPMA method was employed for protein secondary structure prediction (Geourjon and Deléage, 1995). The three-dimensional structure of the important protein was predicted using the online tool SWISS-MODEL (Waterhouse et al., 2018), and protein-protein docking analysis was conducted using the AlphaFold3 server (Abramson et al., 2024).

### Detection of conserved motifs, gene structure, and domains

The protein-conserved motifs of the identified watermelon *ClC4H* gene family were analyzed using the MEME online database (accessed on January 15, 2026) (Bailey et al., 2009). The motif length was set to 6 to 200 amino acids, and the number of motifs was set to 10. The ClC4H protein conserved domain prediction was conducted through the conserved domain (CD)-Search function on NCBI online (Yang et al., 2020). By integrating the complete genome sequence of Watermelon (97103) v2 and annotation file information, the ‘Gene structure view’ function of TBtools-II (v2.3.60, upgraded software) (Chen et al., 2023) was used to display the *ClC4H* gene structure, protein-conserved motifs, and protein functional domain prediction information.

### Phylogenetic evolution of the *C4H* gene family among different species

The C4H protein sequences of *Arabidopsis thaliana* were downloaded from the genetic and molecular biology database of The Arabidopsis Information Resource (TAIR) (Reiser et al., 2024). The C4H protein sequences of *Cucumis melo*, *Cucumis sativus*, *Cucurbita maxima*, *Cucurbita pepo* subsp. pepo, *Cucurbita moschata*, *Glycine max*, and *Nicotiana tabacum* were obtained from the freely accessed website database of the National Center for Biotechnology Information (NCBI). The multiple protein sequence alignments of the *C4H* gene family among different selected species were analyzed using the Align by MUSCLE function of MEGA software (v7.0) (Kumar et al., 2016). The best-fitting amino acid substitution model was determined using a model test based on the Bayesian Information Criterion (BIC) in MEGA software. The Jones-Taylor-Thornton (JTT) model with uniform rates among sites was selected as the optimal model. A maximum likelihood (ML) phylogenetic tree was reconstructed using the Nearest-Neighbor-Interchange (NNI) heuristic method. The initial tree was generated automatically (default NJ/MP). Branch support was evaluated with 1,000 bootstrap replicates. All other parameters were kept as the default.

### Extraction of promoter trans-acting element

The C4H protein sequences of *Arabidopsis thaliana* were downloaded from the genetic database. The promoter region upstream of the CDS of 2000 bp was extracted using the Gtf/Gff3 Sequences Extractor function of the upgraded version of TBtools-II (v2.3.60, upgraded software) (Chen et al., 2023). The cis-acting elements were predicted using the “Search for CARE” function on the PlantCARE online website (Lescot et al., 2002).

### Subcellular localization and interaction network analysis of ClC4H protein

The construction of the subcellular localization vector (pBWA(V)H2STMV-ClC4H1) for the ClC4H1 protein and the transient expression experiment in tobacco leaves were carried out by Beryuan Biotechnology (Wuhan Beryuan Biotechnology Co., Ltd.). The injection of tobacco leaves and confocal observation were conducted according to the transient expression method established in a previous study (Sparkes et al., 2006). The protein interaction network for ClC4H1 was predicted using the STRING database (PPI), and an analysis of the functions and biological processes of the interacting proteins was performed (Szklarczyk et al., 2023).

### Expression verification of C4H and interacting genes at the pericarp developmental stage

The expression levels of *C4H* gene family members and ClC4H-interacting proteins were searched for values of FPKM (Fragments Per Kilobase of transcript per Million mapped reads) by using the uploaded transcriptome database of pericarp tissues of self-crossed watermelon lines (WRH and WRS). The gene expression data of pericarp tissue development at different developmental stages were sorted and visualized using the HeatMap function of the latest software of TBtools-II (v2.3.60) (Chen et al., 2023). The expression levels of genes in different developmental stages of the same self-crossing line material were standardized, the Euclidean distance clustering was selected by the distance method, the complete-linkage clustering method was selected as the clustering analysis method, and the system tree diagram was selected as the branch form.

### Determination of lignin content, activity of C4H enzyme, and rind hardness evaluation

The fresh pericarp samples of watermelon fruits were selected, the outer epidermis layer was removed, and the HCl-pyrogallol staining method was used to stain the stone cell layer (Dyckmans et al., 2010). The thick pericarp sample (1 cm in size) was cut using a custom ring knife (diameter d = 8 cm) to cut 1 cm-thick pericarp samples. After tissue disruption and grinding, the 60% sulfuric acid method is used to determine the microscopic analysis (Mansell et al., 1974).

In brief, 1 g of pericarp material was put in liquid nitrogen and ground; then 0.1 g was taken and put in a 2 ml pre-cooled centrifuge tube. Then, the mercaptoacetic acid method [46] was used to determine the lignin content in the pericarp of the watermelon fruit. Then, 0.5 g of fresh watermelon pericarp sample was taken, and 2 mL of pre-cooled C_4_H lysis buffer was immediately added and ground. The specific determination method can be found in the C4H activity detection kit manual of Regen Biotechnology Company (Sun, 2024). The pericarp hardness of rind samples was tested by measurement of puncture using the TA-XT*plusC* texture analyzer (SMS, Stable Micro System Division, U.K.) with a 2 mm (P/2) probe (Yang et al., 2023).

### Expression validation of key gene

A total of 1 g of sample from leaf, stem, and root tissues was obtained from the young seedlings of WRH and WRS materials at the 5th true leaf stage, and the fruit rind tissues were obtained at the second pistil position after pollination for 21 days. Total RNA was isolated from subsequent frozen samples using the TRIzol method (Yang et al., 2023). The expression trend of the *ClC4H1* gene and other interacting genes was checked in all of the sampled tissues, as well as in the fruit rind tissues, by using three biological replicates and three technical replicates of data (Livak and Schmittgen, 2001). The primers used for qRT-PCR are listed in **Supplementary Table 1**. The internal reference primers are selected as the *ClYLS8*. The specific parameters of the qRT-PCR method refer to the previously reported method (Yang et al., 2023).

### Data analysis

The statistical data analysis was performed using the software GraphPad Prism v11.0.1. Comparisons among different growth periods (1, 14, 28 DAP) were conducted using the independent sample t-test, and the Welch correction was applied (not assuming equal variances of the two populations). Asterisks, * indicate P value < 0.05 and ** indicate P value < 0.005, respectively.

## Results

### Analysis for characteristics of identified *C4H* genes and proteins

We employed a keyword search method within the Cucurbitaceae database, and the HMMER search technique, along with the homologous protein BLASTP search method, was used to identify 6, 7, and 5 putative genes, respectively. A total of 4 candidate *ClC4H* genes were ultimately selected through further functional domain analysis, which were designated as *ClC4H1–4* based on the locations of these four *C4H* genes across the genome-wide chromosomes of watermelon. The relevant details, including corresponding numbers, chromosome positions, gene start and end positions, coding region length, CDS length, exon number, and specific information regarding the characteristics of the encoded proteins, are presented in **Table 1**.

**Table 1:** Physicochemical properties and molecular characterization of the four identified *ClC4H* genes and their encoded proteins in watermelon.

Gene symbol	Gene ID	Chr	Starting point	End position	Length of coding region (bp)	Length of CDS/bp	Number of exons	Coding protein characteristics					
								Amino acid length/aa	MW/kD	PI	Secondary structure characteristics		
											α-helix(%)	β-sheet(%)	Random coil (%)
*ClC4H1*	*Cla97C02G044950*	2	33091905	33095511	3607	1518	3	505	57.96	9.10	47.92	11.49	40.59
*ClC4H2*	*Cla97C03G050850*	3	96292	98229	1938	1518	3	505	58.30	8.53	48.12	12.08	39.80
*ClC4H3*	*Cla97C11G217460*	11	21656102	21657789	1688	1596	2	531	60.72	8.62	51.22	10.92	37.85
*ClC4H4*	*Cla97C11G217490*	11	21673115	21674866	1752	1608	2	535	61.01	8.78	50.47	10.28	39.25

The members of the ClC4H family, *ClC4H1-4*, seemed to be distributed across chromosomes 2, 3, and 11. Mainly, there was only 1 gene located on chromosomes 2 and 3, while 2 genes were found on chromosome 11. The associated coding regions length ranges from 1688 to 3607 bp; the CDS regions spanned between 1518 and 1608 bp, and each region contains two exons. The encoded proteins length ranges between 505 and 535 amino acids (aa), and molecular weights (MW) were 57.96 and 61.01 kD (kilo Dalton). All proteins depicted a high isoelectric point (PI > 7.00), indicating a basic and secondary structure of alpha-helices.

### Analysis of the conserved motifs, gene structure, and functional domains of the ClC4H gene family members

**Figure 1:**
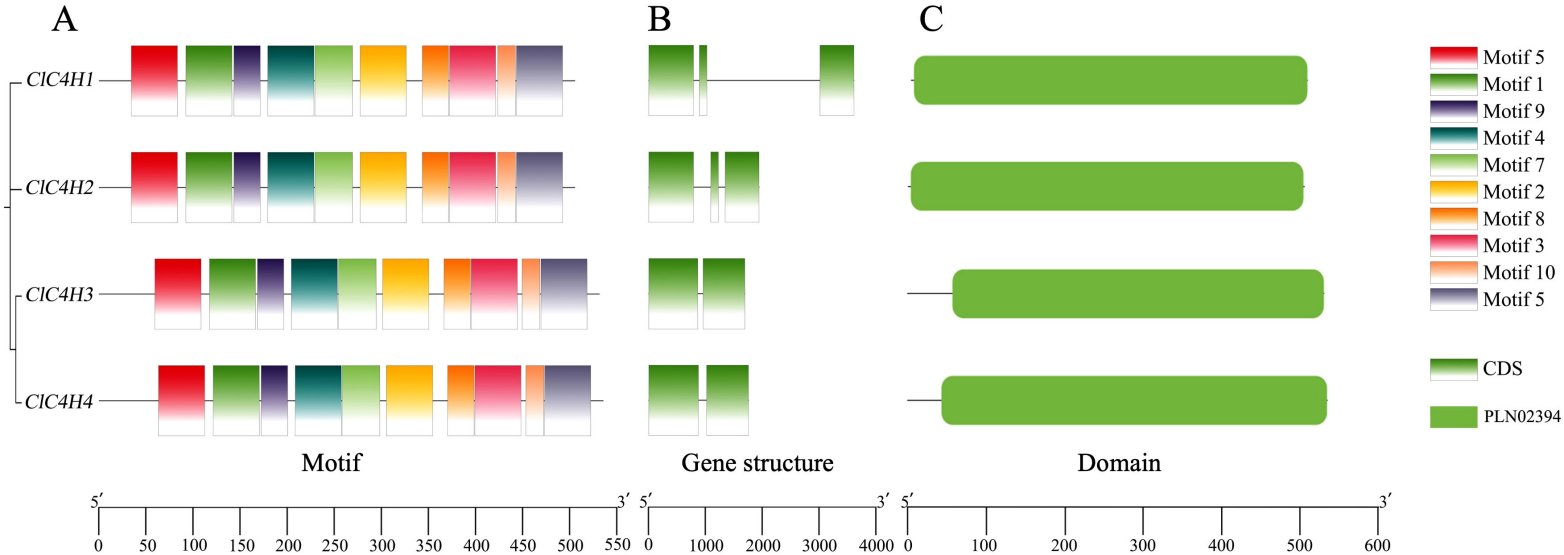
Structural characterization of the *ClC4H* genes. **(A)** Distribution of conserved motifs identified by MEME in ClC4H proteins. Different colored boxes represent distinct motifs. **(B)** Exon–intron organization of *ClC4H* genes; green boxes indicate exons and black lines represent introns. **(C)** Conserved domain architecture of ClC4H proteins showing the cytochrome P450 domain (PLN02394).

To understand the characterization of the *ClC4H* gene family, we studied the usual patterns, the arrangement of exons and introns, and the structure of the formed proteins (**Figure 1**). Motif analysis identified ten conserved motifs (motifs 1–10) among the four ClC4H proteins (ClC4H1–ClC4H4). The order of these motifs was very similar in all the proteins, showing that there is a strong structural similarity within the family (**Figure 1A**). Most of the motifs were found in each protein, with just small differences in their size, suggesting that the ClC4H proteins might have the same biochemical functions while experiencing some small changes in their structure.

The gene structure analysis indicated that the *ClC4H* genes had a similar exon-intron structure (**Figure 1B**). All four identified genes had multiple exons separated by introns, with different lengths of introns. This type of structural conservation is often seen in plant cytochrome P450 genes, indicating that these genes likely evolved from gene duplication events followed by reasonable evolutionary divergence.

The conserved domain analysis revealed that all ClC4H proteins contain the PLN02394 domain, which constitutes part of the catalytic region of cinnamate 4-hydroxylase, a member of the cytochrome P450 monooxygenase superfamily (**Figure 1C**). This domain spans a substantial portion of the protein sequence in each family member, indicating that the essential catalytic region is highly conserved. The consistent presence of this domain in ClC4H proteins strongly suggests that these proteins perform similar functions in the phenylpropanoid biosynthetic pathway, specifically by catalyzing the hydroxylation of cinnamic acid.

Overall, the structural similarities of the conserved regions, consistent exon–intron arrangements, and shared catalytic domain indicate that the *ClC4H* gene family has been conserved throughout evolution and likely contributes significantly to plant secondary metabolism and phenylpropanoid biosynthesis.

### Analysis of conserved motif composition and structural features of ClC4H proteins

**Figure 2:**
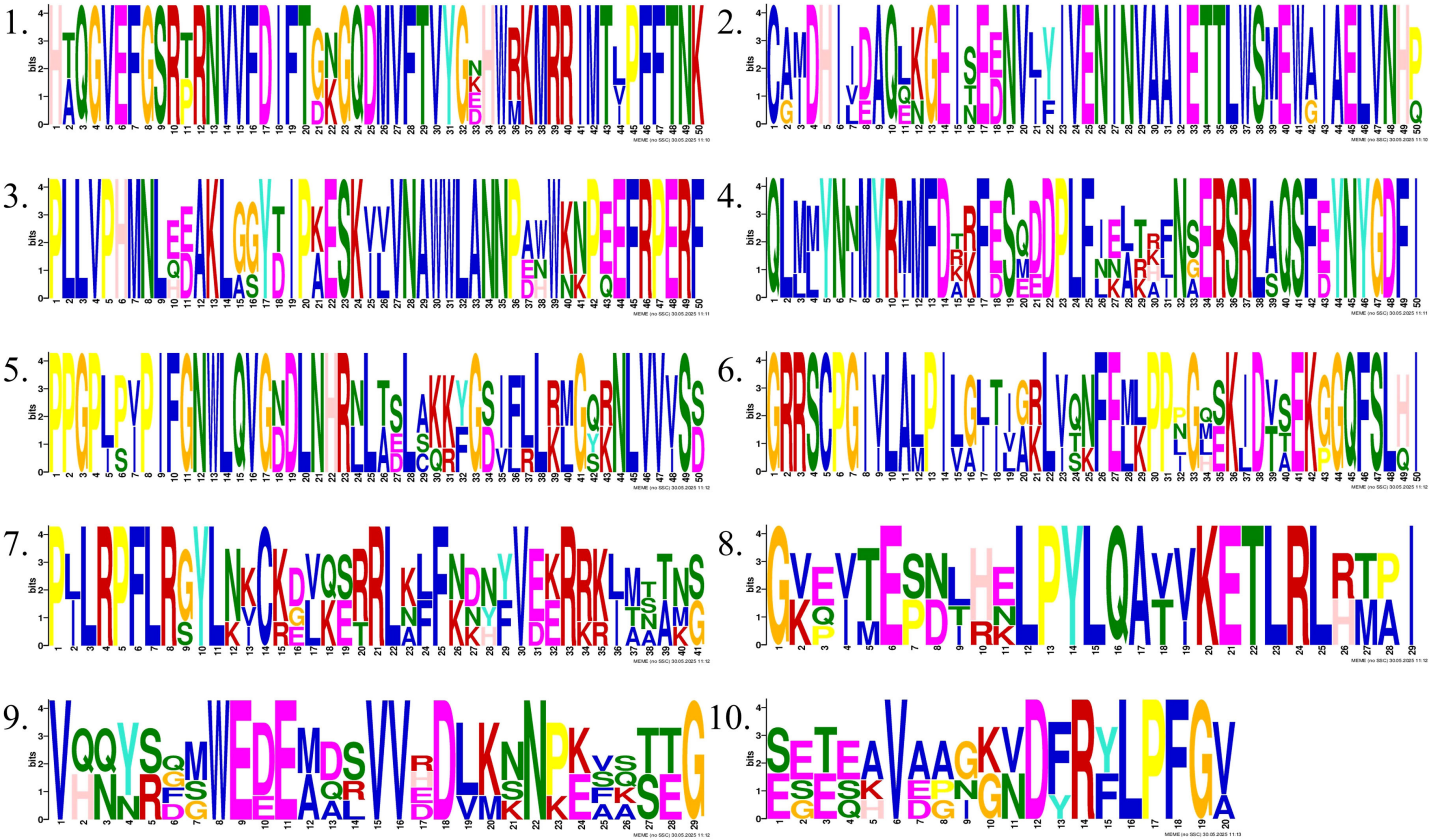
Conserved motif sequence logos of the ClC4H proteins, representing the amino acid conservation of the identified motifs (motifs 1–10) in the ClC4H proteins.

To further elucidate the structural conservation of the ClC4H gene family, conserved motif patterns within the encoded proteins were examined using sequence logo analysis (**Figure 2**). A total of ten conserved motifs (motifs 1–10) were identified among the four ClC4H proteins. The sequence logos illustrate amino acid conservation and positional frequency within each motif, with the height of individual residues reflecting their relative conservation across protein sequences. Several motifs exhibited highly conserved residues, particularly hydrophobic and catalytically relevant amino acids, suggesting their functional importance in maintaining the structural integrity of the protein.

Notably, motifs exhibited strong conservation in the specific amino acid positions, indicating evolutionary constraints associated with the catalytic activity of cinnamate 4-hydroxylase enzymes. For instance, motifs such as Motif 1, Motif 4, and Motif 7 contained multiple conserved residues that are characteristic of cytochrome P450 monooxygenases, which are essential for substrate binding and catalytic function. The high degree of conservation across these motifs suggests that the ClC4H proteins maintain a conserved enzymatic framework necessary for their biological roles.

The presence of conserved amino acid signatures within these motifs further supports the classification of the identified proteins as members of the cinnamate 4-hydroxylase (C4H) family, which plays a key role in the phenylpropanoid biosynthetic pathway. These conserved motifs likely contribute to critical structural elements, e.g., heme-binding regions, substrate recognition sites, and catalytic residues, which are typical features of cytochrome P450 cinnamate 4-hydroxylase enzymes.

Overall, the strong conservation of motif sequences across all ClC4H proteins highlights the functional stability and evolutionary conservation of this gene family. Such conserved structural features suggest that the ClC4H proteins likely retain similar enzymatic functions, contributing to phenylpropanoid metabolism and related physiological processes in plants.

### Analysis for phylogenetic relationships and synteny of *C4H* genes

**Figure 3:**
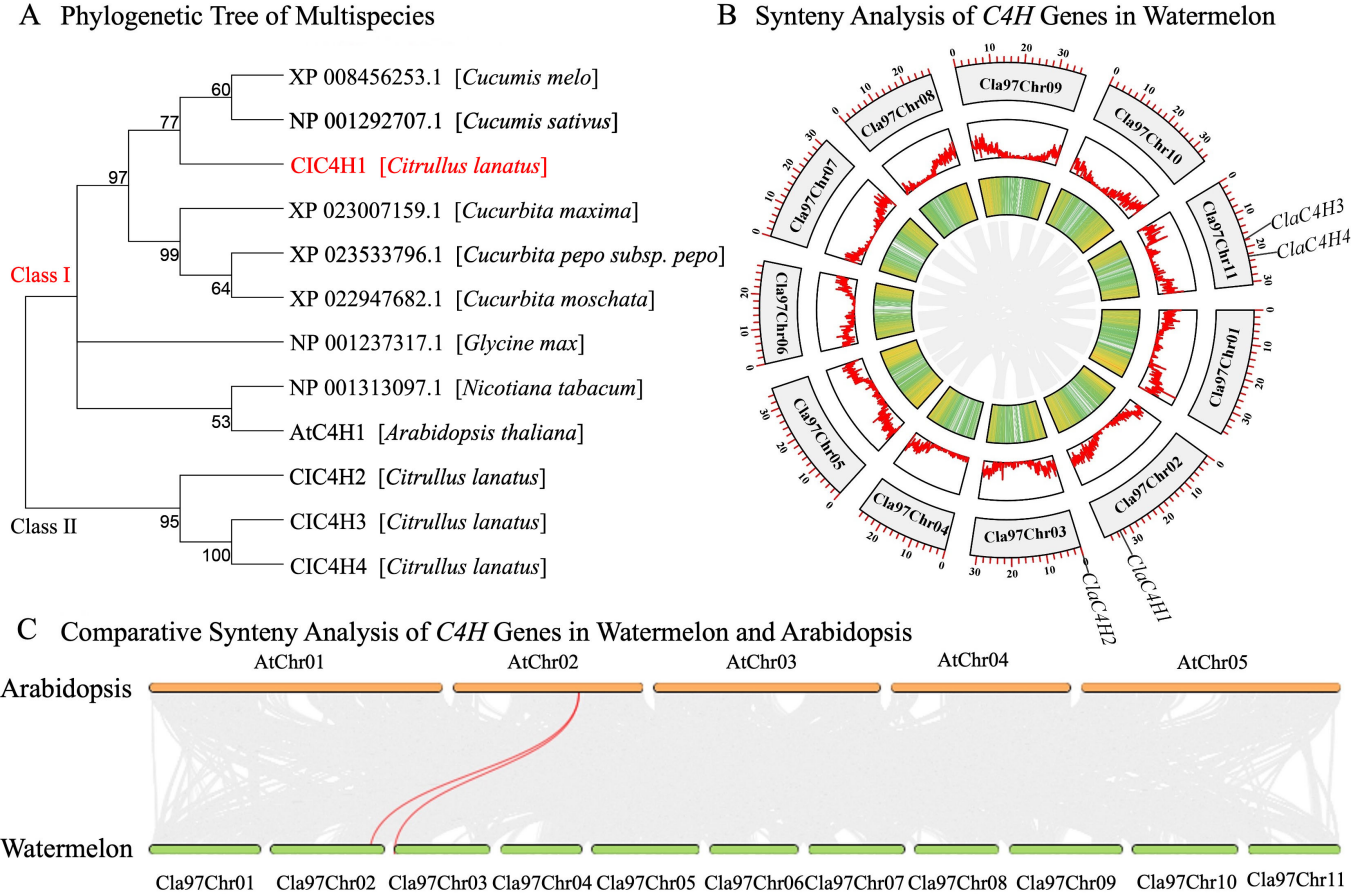
Phylogenetic association and syntenic analysis of *C4H* genes across watermelon and other plant species, highlighting the major divergence by clades. **(A)** Phylogenetic relationships of *C4H* proteins, showing their separation into two major classes (Class I and Class II). **(B)** Chromosomal localization and intra-genomic synteny of *C4H* genes in watermelon. **(C)** Comparative syntenic relationships of C4H genes between watermelon and *Arabidopsis thaliana*, with red lines representing conserved orthologous gene pairs.

The phylogenetic relationship analysis across selected species detected that the members of the *C4H* gene family in *Citrullus lanatus* are basically classified into two major classes (**Figure 3A**). The protein sequences of the Class I branches (ClC4H1) exhibited greater similarity to those found in *Arabidopsis thaliana*, *Glycine max*, *Nicotiana tabacum*, and various cucurbitaceous crops. Within this class, ClC4H1 shows the closest relationship with *Cucumis melo* and *Cucumis sativus*. Class II consists of three watermelon-specific paralogs (ClC4H2, ClC4H3, and ClC4H4). Notably, ClC4H3 and ClC4H4 form a well-supported monophyletic clade (bootstrap = 100%), indicating a very close evolutionary relationship, while ClC4H2 is basal to this pair. All three Class II members are evolutionarily distant from ClC4H1, suggesting they represent a unique, watermelon-specific C4H lineage derived from species-specific gene duplication events.

Gene collinearity analysis illustrated additional insights into the possible evolution and origin of identified genes, and the *ClC4H* genes confer the distribution across the chromosomes. In **Figure 3B**, different colors indicate gene density from various chromosomes, facilitating an understanding of the positional distribution and arrangement relationships of genes within the genome. However, the analysis depicted no significant collinearity within the ClC4H gene family. However, two pairs of collinear gene pairs (**Figure 3C**) were detected when compared to *Arabidopsis thaliana*, specifically AtC4H1 with ClC4H1 and AtC4H1 with ClC4H2. This may imply that these two genes are relatively conserved throughout evolution. In contrast, ClC4H3 and ClC4H4 lack syntenic counterparts in *Arabidopsis thaliana*, suggesting they may have originated from watermelon-specific duplication events after the divergence of cucurbits and brassicas. However, the conserved motif composition and gene structure characteristics observed in all four ClC4H proteins further confirm the functional conservation of these proteins in the biosynthesis pathway of phenylpropanoid compounds.

### Analysis of cis-acting elements in the gene promoter

**Figure 4:**
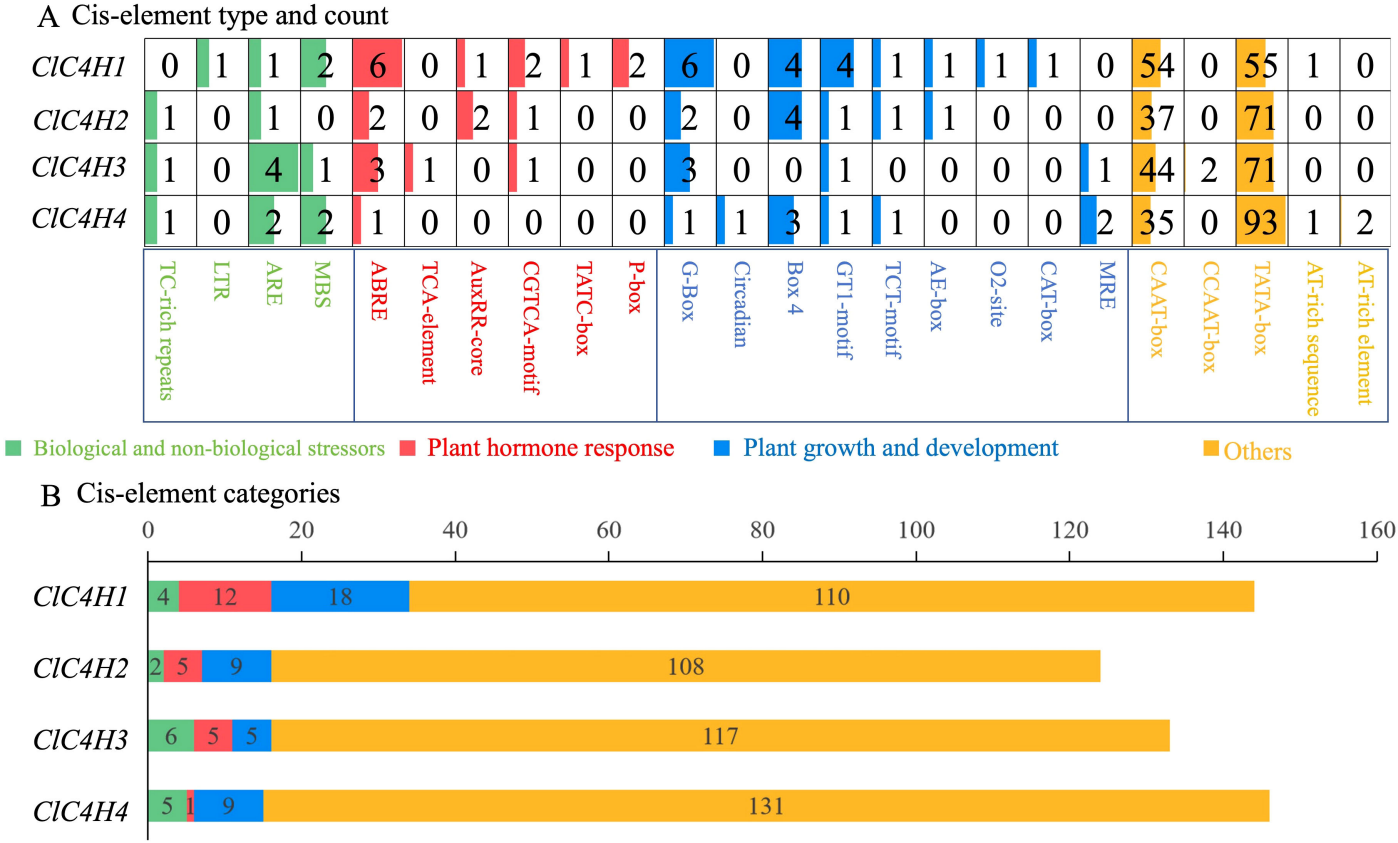
Composition and distribution of predicted cis-acting regulatory elements within the promoter regions of *ClC4H* genes. **(A)** Types and copy numbers of predicted cis-elements. **(B)** Functional categorization and distribution of cis-elements across *ClC4H* promoters.

The analysis of the cis-regulatory elements in the promoter regions of the members of the *ClC4H* gene family indicates that the expression regulatory network is complex, mainly involving basic transcription, environmental stress responses, plant hormone signaling, and light regulation, among others. As shown in **Figures 4A, B**, all member promoters contain a large number of core promoter elements, such as the TATA box (accounting for 62.23% of all elements) and the CAAT box (accounting for 36.48%). These elements are the basis for the recognition and binding of the transcription machinery, mainly determining the basal transcription level and the accuracy of initiation of the gene, and non-specifically participating in the growth and development process.

In addition, other environment-specific regulatory elements have also been identified, such as the core light response element (G-box/GT1-motif), anaerobic response element (ARE), etc. The hormone response elements are mainly abscisic acid response elements (ABRE) and also include the auxin response core element (AuxRR-core), the jasmonic acid methyl ester response element (CGTCA-motif), and the auxin response core element (AuxRR-core). It is speculated that they may regulate various physiological processes through hormone regulation. The expression of the members of the *ClC4H* gene family appears to be associated with basic promoter transcription elements and may be influenced by a complex network involving abiotic stress signals, various plant hormones, and light signals. This suggests that this gene family might play a role in plant adaptation to environmental changes and in the regulation of growth and development.

### Analysis of *C4H* gene expression profiling and interactive genes during different rind Development stages of watermelon

**Figure 5:**
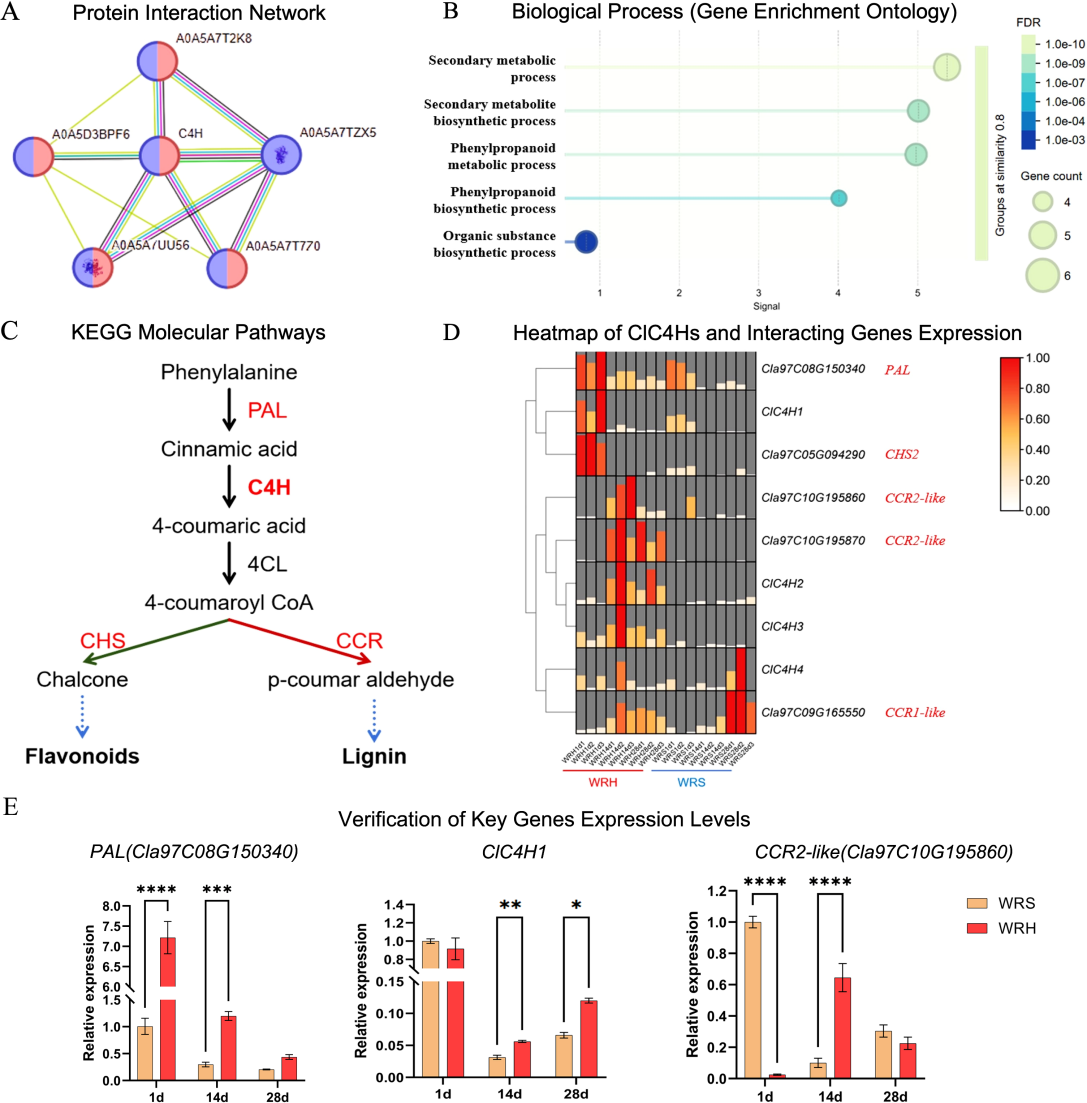
Integrated analysis of protein network, functional enrichment, and spatiotemporal expression profiling of *ClC4H* genes in phenylpropanoid biosynthesis. **(A)** Protein interaction network. **(B)** GO biological process enrichment. **(C)** KEGG phenylpropanoid pathway. **(D)** Expression heatmap of *ClC4H* and interacting genes. **(E)** qRT-PCR validation of key genes. * (Significant): (p < 0.05), ** (Very significant): (p < 0.01), *** (Extremely significant): (p < 0.001), **** (Highly extremely significant): (p < 0.0001).

It appeared that the ClC4H1 protein and other interacting proteins may be primarily involved in lignin synthesis-related pathways, which are responsible for the biosynthesis of secondary metabolites and phenylpropanoid compounds (**Figure 5A**). The PPI interaction network predicted that the network contains 6 protein nodes and 12 interactive associations. The GO functional enrichment analysis showed that the 6 proteins mainly participate in two aspects: biological process and molecular function (**Figure 5B**, **Table 2**).

**Table 2:** Functional annotation and gene ontology (GO) enrichment of *ClC4H1* and its predicted interacting proteins in the phenylpropanoid pathway.

Melon gene	Homologous gene in watermelon	Annotation
C4H	ClC4H1	Cytochrome P450 family cinnamate 4-hydroxylase
A0A5A7TZX5	Cla97C05G094290.1	Chalcone synthase 2 (CHS2)
A0A5D3BPF6	Cla97C08G150340.1	Phenylalanine ammonia-lyase (PAL)
A0A5A7T2K8	Cla97C10G195860.1	Cinnamoyl-CoA reductase 2-like (CCR2-like)
A0A5A7UU56	Cla97C09G165550.1	Cinnamoyl-CoA reductase 1-like (CCR1-like)
A0A5A7T7Z0	Cla97C10G195870.1	Cinnamoyl-CoA reductase 2-like (CCR2-like)

In the biological process category, all six proteins were predicted to be involved in secondary metabolic processes, and their molecular functions were all characterized by catalytic activity. The core function of ClC4H1 appeared to be mainly catalytic and oxidoreductase activity, which is similar to the predicted functions of the three proteins of CCR2-like (*Cla97C10G195860.1* and *Cla97C10G195870.1*) and CCR1-like (*Cla97C09G165550.1*). While CHS2 (*Cla97C05G094290.1*) and PAL (*Cla97C08G150340.1*) only show catalytic activity and lack specific oxidoreductase activity functions, indicating that the four proteins represented by *ClC4H1* are likely to be the core enzymes responsible for the key oxidation-reduction steps, the other two proteins may undertake other types of catalytic functions, working together to complete the complex synthesis of secondary metabolites. The KEGG analysis results (**Figure 5C**) showed that all 6 proteins are involved in the biosynthesis of secondary metabolites, except for the *CHS2* (*Cla97C05G094290.1*) protein, which is involved in the biosynthesis of phenylpropanoid compounds.

We also examined the gene expression patterns in rind tissues of both watermelon materials (WRH with high lignin and WRS with low lignin) at different time points after pollination (1 day, 14 days, and 28 days) and evaluated the potential regulatory roles of the *C4H* gene family and its interacting genes involved in lignin biosynthesis. As shown in the heatmap analysis (**Figure 5D**), the C4H family genes and their interacting genes showed significant spatiotemporal-specific expression patterns and were highly correlated with the lignin content phenotype of the materials. In the early development stage of WRH materials (1 day after pollination, DAP), PAL (*Cla97C08G150340*), ClC4H1, and CHS2 (*Cla97C05G094290*) showed significantly high transcription levels (red annotation), while in all time points of WRS materials, they maintained low expression, indicating that this gene module was the core promoter of early lignin synthesis in WRH materials, directly driving the initiation of the high lignin phenotype. When fruits entered in the mid-development stage (14 days after pollination), the CCR2-like (*Cla97C10G195870*), *ClC4H2*, and CCR2-like (*Cla97C10G195860*) continued to express at high levels in the WRH materials, further strengthening the process of lignin accumulation; in contrast, the expression levels of these genes in the WRS materials remained at a relatively low level, revealing that the mid-stage expression differences are the key nodes leading to the differentiation of lignin content between the two types of materials.

Heat map cluster analysis of ClC4H gene and interacting genes further revealed the functional synergy of the genes, jointly regulating lignin synthesis (**Figure 5D**). Among them, PAL (*Cla97C08G150340*), *ClC4H1*, and *CHS2* (*Cla97C05G094290*) clustered together, These proteins may jointly participate in the initiation of early lignin synthesis in WRH; CCR2-like (*Cla97C10G195860*), CCR2-like (*Cla97C10G195870*), *ClC4H2*, and *ClC4H3* clustered into the second category, possibly regulating the continuous accumulation of lignin in WRH during the mid-stage; while *ClC4H4* and CCR1-like (*Cla97C09G165550*) clustered into the third category, significantly upregulated only in the late stage (14 days after pollination, DAP) of the WRS materials. Further, the relative expression levels of key genes (**Figure 5E**), *PAL* (*Cla97C08g150340*), *ClC4H1*, and *CCR2-like* (*Cla97C10G195860*), showed strong expression verification between developmental stages (1, 4, and 28 DAP). It is speculated that inhibiting lignin synthesis or regulating alternative pathways of cell wall metabolism ultimately leads to the low lignin phenotype of the WRS materials, but their specific functions still require further experimental verification. The temporal and spatial-specific expression of these functional modules jointly shaped the dynamic differences in lignin content between the WRH and WRS varieties.

### Analysis of histological and physiological indexes

**Figure 6:**
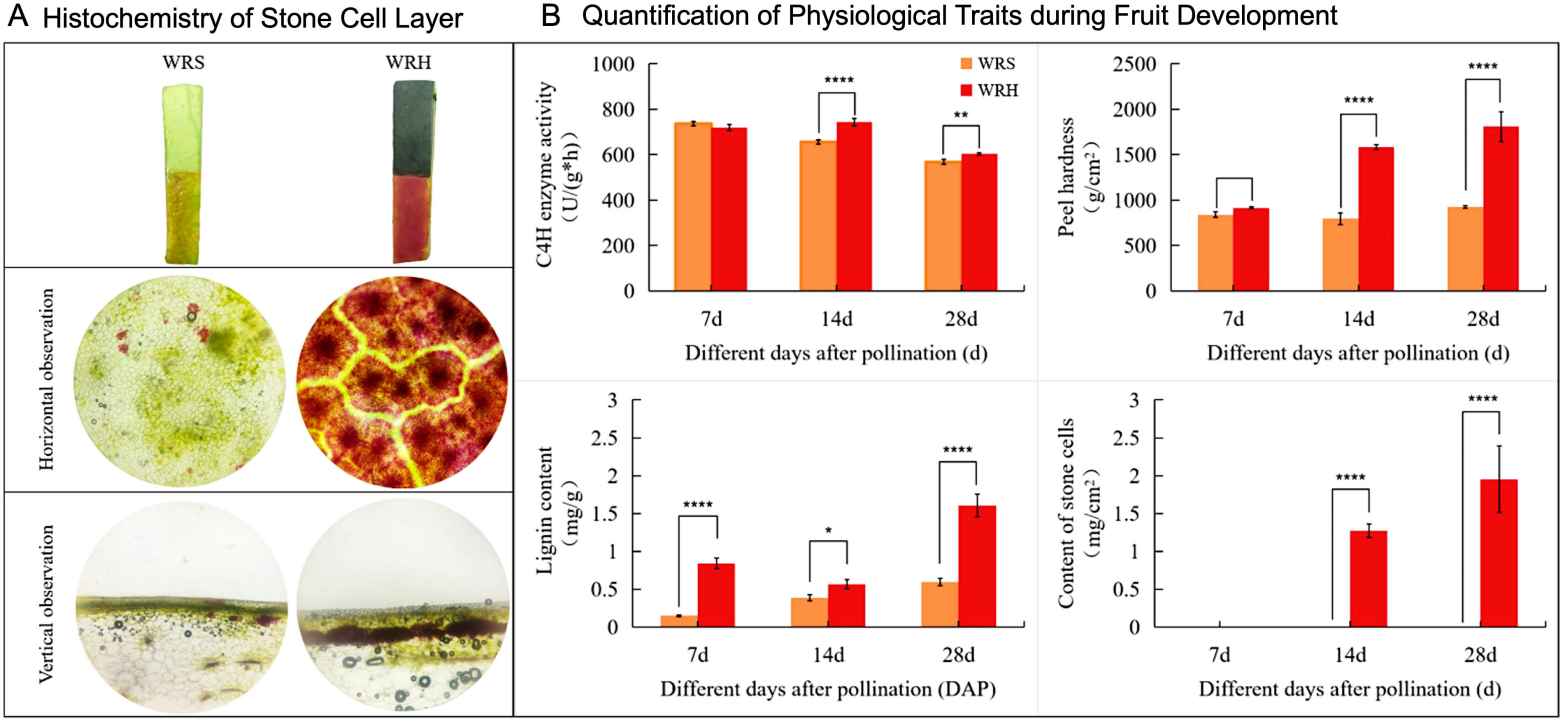
Comparative analysis of lignin accumulation, stone cell density, and C4H enzymatic activity between fruit rinds of contrasted watermelon materials (WRH and WRS). **(A)** Horizontal and vertical histochemical observations of the stone cell layer. **(B)** Quantification of C4H enzyme activity, peel hardness, lignin content, and stone cell accumulation at 7, 14, and 28 days after pollination. Data are presented as mean ± SD. Asterisks (*, **, ****) indicate significant differences at P < 0.05, P < 0.01, and P < 0.0001, respectively.

To verify the molecular-level predictions, we further conducted the histological, physiological, and biochemical index evaluations between the rind tissues of contrasted watermelon materials (WRS and WRH), and significant variations were noticed. The transverse sections (red reticular lignin staining) and longitudinal sections (dark stone cell layer) of WRH (**Figure 6A**) showed that the thickness of the stone cell layer and the lignin accumulation were significantly higher than those of WRS, directly reflecting that the stone cell layer of WRH was more developed. The WRH variety had significantly higher C4H enzyme activity, rind hardness, lignin content, and stone cell content than WRS, and the index values continued to increase with the development time of 28 days (**Figure 6B**).

Specifically, in the WRH variety, higher expression levels of *ClC4H1/2/3* were associated with increased C4H enzyme activity, greater lignin and stone cell accumulation, and enhanced fruit rind hardness compared with the WRS variety. These coordinated changes were evident across all three developmental stages (7, 14, and 28 DAP) as shown in **Figure 6B**, and corresponded to the more developed stone cell layer observed in the histological sections of WRH (**Figure 6A**).

### Analysis of expression pattern and protein model of ClC4H1

**Figure 7:**
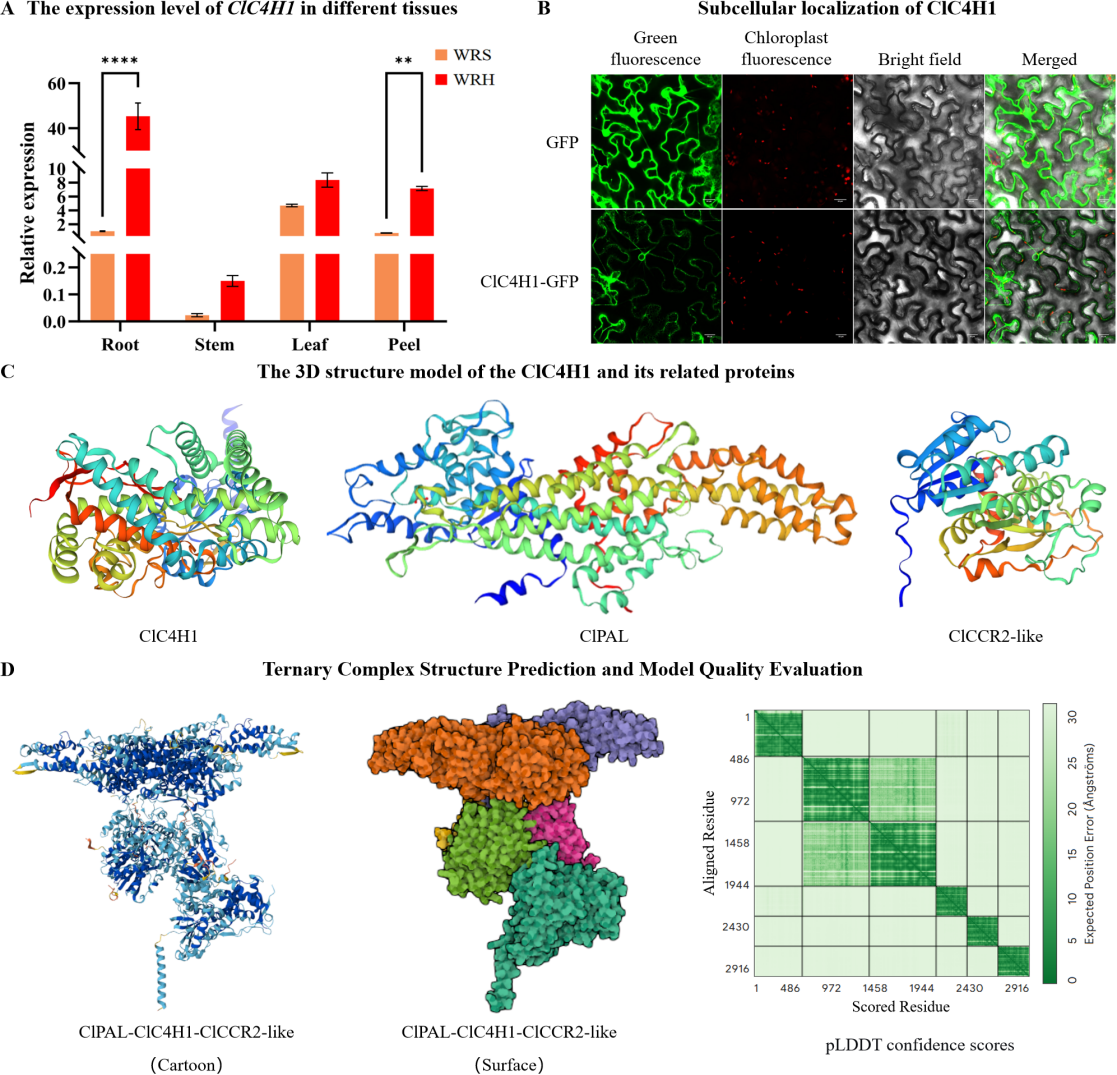
Functional verification, localization, and structural analysis of ClC4H1 as a key component of phenylpropanoid-associated protein complexes. **(A)** Tissue-specific expression of *ClC4H1* in WRS and WRH. **(B)** Subcellular localization of ClC4H1-GFP in tobacco leaf cells. **(C)** Monomeric 3D structure models of ClC4H1, ClPAL, and ClCCR2-like. **(D)** Predicted surface structures and confidence evaluation matrix of the ternary ClPAL-ClC_4_H_1_-ClCCR_2_-like complex. Data are presented as mean ± SD (*n* = 3). Significances are shown as ** (P < 0.01) and **** (P < 0.0001).

The tissue-specific expression of the *ClC4H1* gene was analyzed through qRT-PCR verification in roots, stems, leaves, and pericarp tissue; however, a higher expression level was found in root tissues compared to the stems. Overall, the *ClC4H1* gene expression level was observed to be higher in the pericarp of WRH material (with higher lignin content) than that in the WRS materials. Moreover, its expression was also higher in roots, stems, and pericarp of leaves compared to WRS materials (**Figure 7A**).

The subcellular localization experiment indicated that the ClC4H1 protein was mainly located on the endoplasmic reticulum. As shown in **Figure 7B**, its green fluorescence distribution was in a net-like, tubular interwoven structure, with fuzzy signals surrounding the nucleus and extending to the edge of the cytoplasm, forming a dense network, which is a typical morphological feature of endoplasmic reticulum localization. Moreover, the red fluorescence was scattered in a dot-like distribution in the cytoplasm, without obvious co-localization with the green fluorescence signal, thereby confirming the specificity of its endoplasmic reticulum localization.

To explore potential protein-protein interactions (PPI), the ClC4H1 protein was homologously modeled with related proteins (ClPAL and ClCCR2-like) using SWISS-MODEL (**Figure 7C**). Subsequently, through the calculation of the AlphaFold3 server, we obtained the predicted model of the ternary complex. The template modeling score (pTM) of the prediction model was 0.48, the interface template modeling score (ipTM) was 0.33, and the comprehensive score (pTM + ipTM) was 0.81, which exceeds the suggested interaction threshold of 0.75. Based on this computational prediction, ClC4H1, ClPAL, and ClCCR2-like may have a potential tendency to interact with each other (**Figure 7D**).

## Discussion

Lignification is the biological process that strengthens plant tissues, enables upright growth, provides structural support to the xylem, and acts as a defensive barrier against pathogens. In horticultural crops, e.g., watermelon (*Citrullus lanatus*), the degree of lignification in the stone cell layer of fruit rind is a primary determinant of rind hardness, which directly impacts post-harvest storage stability, transportation tolerance, and overall commodity value (Xu et al., 2012). Cinnamate 4-hydroxylase (C4H) acts as a critical rate-limiting “hub” among the key enzymes in the phenylpropanoid metabolic pathway, triggering the carbon flow toward the cell wall-associated biosynthesis of lignin (Aravena-Calvo et al., 2024). While the role of *C4H* in regulating stone cell formation has been documented in species such as pear and peach, its genomic organization and functional contribution to watermelon fruit quality remain largely unexplored.

In the previous study of Yang et al. (2023), the molecular genetic breeding-based linkage mapping experiment elucidated the stable genomic regions affecting the rind hardness trait in watermelon grown across multiple years. However, in the current study, we conducted the first comprehensive and systematic bioinformatic approach for genome-wide characterization of the C4H gene family for genomic and functional dissection of key regulators of rind texture in the *Citrullus lanatus*. A total of 4 distinct members (ClC4H1-4) with different structural characterization across three different chromosomal positions (**Table 1**) collectively showed the modulation of lignin deposition through specialized spatiotemporal expression patterns. The number of identified genes (*ClC4H1-4*) is similar to that of the orchid species (Kaur et al., 2025) and the *Arabidopsis thaliana* species and the soybean species (Khatri et al., 2023). It has also been reported that C4H, as a member of the cytochrome P450 CYP73A subfamily, typically exists in plants as a smaller gene family, which may harbor primary function as a key rate-limiting enzyme in the early stage of phenylpropanoid metabolism. Its encoding gene is subject to strong functional constraints during evolution to maintain the stability of the core metabolic pathway (Winkel-Shirley, 2001; Hamberger et al., 2011).

However, despite the limited number of members, the ClC4H genes in watermelon show significant differentiation in gene structure (with 2–3 exons) (**Figure 1**) and system evolution, clustering into two branches (**Figure 3**): Class I (ClC4H1) and Class II (ClC4H2, ClC4H3, and ClC4H4), suggesting that they may have undergone function-specific evolution. ClC4H1 has the highest homology with AtC4H in Arabidopsis and C4H homologs in closely related cucurbit species (melon and cucumber) (**Figure 3**), suggesting that they may undertake the conserved function of the core pathway of phenylpropanoid metabolism, especially lignin synthesis (Dixon and Paiva, 1995; Winkel-Shirley, 2001). Herein, the Class II clade, comprised of ClC4H2, ClC4H3, and ClC4H4, seemed relatively independent in the evolutionary tree and had a distant genetic relationship with the members of Class I (**Figure 3**), proposing that they may have evolved certain specific evolutionary functions in watermelon. This branch has also been reported in other species, such as soybean, where different evolutionary branches of C4H members may have differentiated enzymatic properties or expression regulation patterns (Khatri et al., 2023). The promoter analysis (**Figure 4**) shows that all *ClC4H* genes contain abundant cis-regulatory elements related to light response (G-box), hormone response (abscisic acid ABRE, auxin AuxRR-core, and jasmonic acid methyl CGTCA-motif), and abiotic stress response (anaerobic ARE), further supporting the diversity of the gene function and its expression being precisely regulated by complex environmental signals and developmental programs (Kaur et al., 2025; Zhang et al., 2024; Zeng et al., 2025). This provides the significant transcriptional-level regulatory basis for the *C4H* genes to respond to different external and internal signals and finely control the allocation of carbon flow to different phenylpropanoid derivatives (such as lignin, flavonoids, and defensive phenols).

The expression profile results of this study (**Figure 5**) provide direct evidence for revealing the role of *ClC4H* genes in watermelon fruit development, especially in the process of fruit rind lignification. In high lignin materials (WRH), *ClC4H1* showed high expression 1 day after pollination (early stage) and was co-expressed with the upstream gene phenylalanine ammonia-lyase (PAL) and downstream genes that may compete for carbon flow (chalcone synthase 2, CHS2), forming an early activation module. This is consistent with the previous study findings, where C4H, as a key enzyme downstream of PAL, often showed upregulated expression accompanied by the initiation of lignin synthesis (Ehlting et al., 1999). In fruits such as pears and peaches, the high expression of *C4H* genes has also been confirmed to be significantly positively correlated with stone cell development and lignin deposition (Hu et al., 2011; Li et al., 2020).

Further, it was noticed in our experiment that *ClC4H2* was continuously highly expressed during the mid-development stage (14 days) of WRH material and co-expressed with the cinnamoyl-CoA reductase (CCR2-like) gene (**Figure 5**). This may have dominated the orientation of metabolic flow during the peak period of lignin monomer synthesis, further consolidating the accumulation of lignin. Notably, *ClC4H4* was specifically highly expressed in the late developmental stage (28 days) of low-lignin materials (WRS), and combined with the specificity of its evolutionary branch (Class II). So, it is speculated that it may inhibit or balance the excessive lignin accumulation in the later stage of WRS materials through some negative feedback mechanism or by redirecting the carbon flow to branches other than lignin synthesis (such as the flavonoid pathway). This distinct spatiotemporal expression pattern between high and low lignin materials strongly suggests that *ClC4H1* and *ClC4H2* are the key positive regulatory factors driving the high lignin phenotype of the watermelon rind, while *ClC4H4* may play an inhibitory or balancing role. This discovery not only confirms the crucial role of *C4H* as a carbon flow distribution hub (Ehlting et al., 1999; Winkel-Shirley, 2001) but also provides clear candidate targets for precise regulation of the texture (hardness) of the watermelon rind through molecular means (such as gene editing or overexpression).

Protein interaction network prediction analysis has elevated our research from the single gene expression level to the protein complex and metabolic network level. The PPI prediction results (**Figure 5A**) showed that ClC4H1 interacts with multiple key enzymes of the phenylpropanoid pathway, such as PAL and CCR. This strongly supports the previous viewpoint that C4H, along with upstream enzymes like PAL and downstream enzymes like 4-caffeoyl-CoA ligase (4CL), may form a dynamic multi-enzyme complex on the endoplasmic reticulum membrane surface, namely a “metabolon” (Achnine et al., 2004; Chen et al., 2011). This channeling effect can limit unstable intermediate products to a local microenvironment, achieving efficient and targeted conversion of substrates, avoiding unnecessary diffusion or degradation, and thus finely regulating the distribution of carbon flow between different final products (lignin vs. flavonoids) (Ehlting et al., 1999; Winkel-Shirley, 2001).

In addition, our GO and KEGG enrichment analysis (**Figures 5B, C**) suggested that *ClC4H1* and its predicted interacting proteins are potentially involved in the “biosynthesis of phenylpropanoid compounds” and “biosynthesis of secondary metabolites” pathways, and most of the proteins (ClC4H1 and CCRs) have redox enzyme activity, while PAL and CHS2 exhibit other types of catalytic activity, implying possible complementary and synergistic functions among the enzymes within the putative complex. Further, the heat map analysis (**Figure 5D**) and gene expression patterns (**Figure 5E**) in rind tissues of contrasted watermelons (WRH with high lignin and WRS with low lignin) at different developmental stages after pollination (1, 14, and 28 days) showed significant expression and disclosed the potential regulatory roles of the C4H gene family and its interacting genes involved in lignin biosynthesis, similarly revealed by the endogenous histological quantification (**Figure 6**). Subcellular localization indicated that *ClC4H1* is located in the endoplasmic reticulum (**Figure 7**), which is fully consistent with the previous (Winkel-Shirley, 2001; Achnine et al., 2004), where typical N-terminal transmembrane domain features and membrane-anchored P450 enzyme function of the C4H protein have been stated, providing a structural basis for its participation in the assembly of multi-enzyme complexes on the endoplasmic reticulum membrane.

Therefore, we speculated that *ClC4H1* may act as one of the core coordinators through interactions with enzymes like PAL during the early developmental stage of WRH materials, assembling an efficient lignin precursor synthesis metabolic channel; thereby ensuring the rapid initiation and large-scale accumulation of lignin. This hypothesis requires further verification through yeast two-hybrid, bimolecular fluorescence complementation (BiFC), or co-immunoprecipitation experimental approach.

## Conclusion

Herein, we identified four watermelon *ClC4H* genes across two evolutionarily distinct clades using genome-wide comprehensive bioinformatics analysis. Though the *ClC4H1* and *ClC4H2* seem to promote lignification in hard-rind watermelon with high-lignin content (WRH), the *ClC4H4* depicts a balancing or inhibitory role in soft-rind material with low-lignin content (WRS). Our STRING-based protein-protein interaction network and structural modeling further suggest that ClC4H1 could serve as a metabolic anchor for PAL/CCR complexes, potentially contributing to carbon flux toward lignin biosynthesis. These findings establish a comprehensive framework for understanding the molecular and genetic basis of watermelon rind texture and quality, providing strategic targets for breeding schemes to improve rind texture, shelf life, and transportability.

## Data Availability

Publicly available datasets were analyzed in this study. This data can be found here: https://www.ncbi.nlm.nih.gov/bioproject/?term=PRJNA915415 https://www.ncbi.nlm.nih.gov/bioproject/PRJNA1096440.

## Supplementary material

Supplementary Table 1 Gene primers used for qRT-PCR analysis.
